# “Pupdates” on proteasomal degradation in bacteria

**DOI:** 10.1128/jb.00111-25

**Published:** 2025-06-05

**Authors:** Shoshanna C. Kahne, K. Heran Darwin

**Affiliations:** 1 Department of Microbiology, New York University Grossman School of Medicine 12296, New York, New York, USA; University of Florida, Gainesville, Florida, USA

**Keywords:** *Mycobacterium*, proteasome, pupylation

## Abstract

Proteasomes are multi-subunit proteases found in all domains of life. The central components of proteasomal degradation are conserved, but how proteins are targeted to proteasomes diverges significantly. Despite the vast amount of information learned about how proteasomal degradation is regulated in eukaryotes, much less is known about the regulation of proteasome activity in bacteria. In this minireview, we highlight recent findings revealing how and when specific proteins are targeted to bacterial proteasomes, with a focus on ATP-dependent proteolysis.

## INTRODUCTION

In 1977, the identification of a protein with two amino (N)-termini that was susceptible to lysosome-independent degradation opened the door to a world of protein post-translational modifications (PTMs) and proteolytic machines (1). The discovery of ubiquitin and its role as a tag for degradation by a chambered protease, called the proteasome, exploded into numerous areas of study. Investigation of proteasomal assembly and identification of hundreds of enzymes associated with ubiquitylation, deubiquitylation, and degradation of ubiquitylated proteins have provided insight into almost all aspects of eukaryotic biology (reviewed in reference 2). Although initially thought to be exclusive to eukaryotes, the advent of genome sequencing revealed that all archaea and some bacteria encode proteasome components (3). While many prokaryotes also encode ubiquitin-like proteins (4), for a long time, nothing was known about the roles of these proteins, or proteasomes, in their physiology. More than 30 years after the discovery of ubiquitin, prokaryotic ubiquitin-like protein or “Pup” was found to tag proteins for proteasomal degradation in mycobacteria; however, Pup looks nothing like ubiquitin at the sequence or structural level (5, 6). Nonetheless, the discovery of Pup established the field of protein-on-protein PTMs in bacteria.

Proteasomes and “pupylation” are essential for *Mycobacterium tuberculosis* to cause lethal infections in animals (7–11). Consequently, more than two decades of research have focused on characterizing enzymes required for bacterial proteasome function. Remarkably, all cellular pupylation and “depupylation” require a single ligase and amidase, respectively, and numerous proteins can be pupylated, suggesting additional factors must be required to coordinate degradation. In this minireview, we discuss recent advances in our understanding of how the Pup-proteasome system is regulated and highlight promising areas for further investigation.

## CORE COMPONENTS

Despite their evolutionary distance, bacterial and eukaryotic proteasomes share many structural and functional features. At the most basic level, proteasomes are composed of two stacked, heptameric rings of β-subunits with catalytic activity. These rings are sandwiched between two heptameric rings of α-subunits, forming a cylindrical complex called the 20S core particle “20S CP” (12, 13). To prevent unregulated proteolysis, the N-termini of the α-subunits gate entry into the proteolytic core by blocking the channel at both ends (14). Bacteria typically encode a single α- and single β-subunit (referred to as PrcA and PrcB, respectively), whereas eukaryotic proteasomes contain seven unique α- and seven unique β-subunits, only three of which are proteolytically active (15). Each active β-subunit in eukaryotic proteasomes only cleaves specifically after hydrophobic, basic, or acidic residues (16), while mycobacterial β-subunits can cleave after all three sequence types (17). For more detailed background about proteasome structures, we direct readers to references 18–20.

## REGULATION AT THE CORE

In addition to the 20S CP, degradation requires an “activator,” a multimeric complex that stimulates a conformational change of the proteasome α-subunits, opening the protease core to substrates. Two bacterial proteasome activators (Bpa) have been extensively characterized. Mycobacterial proteasome ATPase (Mpa; or ATPase forming ring-shaped complexes [ARC] in non-mycobacteria) is a homo-hexameric complex that unfolds substrates in an ATP-dependent manner (21–24). Mpa monomers are likely to be in a variety of nucleotide binding states, generating a flexible, gapped ring (24). Along these lines, Kavalchuck et al. showed using a substrate-engaged Mpa-proteasome complex in which Mpa rings establish two conformational states to move a substrate into a proteasome core (25). This work allowed the observation of a spiral “staircase” formation of Mpa in which a substrate is transported toward the proteasome core entrance by a “hand-over-hand” mechanism.

Proteasomes can also degrade proteins in an ATP-independent manner when associated with a different activator, proteasome accessory factor E (PafE) (also known as Bpa). PafE forms homo-dodecamers that mediate ATP-independent proteasomal degradation of unfolded proteins and at least two specific substrates (26–28). Protein disorder is required, but not sufficient, for PafE-mediated degradation in *Mycobacterium smegmatis* (29). Notably, Mpa and PafE promote the degradation of distinct protein subsets in *M. tuberculosis* (26, 30).

A third activator, Cpa, with high sequence and structural similarity to Mpa has also been associated with proteasome function in *M. smegmatis*. The proteome is significantly altered in a *cpa* mutant, which has a growth defect under carbon starvation, but it is unknown if proteomic changes are due to altered degradation, gene expression, or both (31).

An essential feature of PafE and Mpa is a four-amino-acid carboxy (C)-terminal glycine-glutamine-tyrosine-leucine or “GQYL” motif required for opening the proteasome to substrates in mycobacteria (10, 21, 26, 27, 32). Interestingly, despite widespread conservation of this motif, the glutamine is not essential for function (26). Eukaryotic proteasome activators have an analogous C-terminal regulatory motif consisting of a hydrophobic amino acid, tyrosine, plus any amino acid (“HbYX”) that is also required for proteasome activation. It is thought that salt bridges form between the C-terminal motif of activators and a lysine conserved in almost all, if not all, α-subunits (reviewed in reference 33). Consistent with this model, replacement of this lysine with alanine in the *M. tuberculosis* proteasome prohibits PafE-dependent degradation *in vitro* (28).

In both PafE and Mpa, amino acids proximal to the GQYL motif restrict engagement with 20S CPs. Although PafE and proteasomes are active *in vitro*, removal of 5–15 residues preceding the GQYL motif in PafE enhances interaction with the proteasome and proteolytic activity *in vitro* and *in vivo* (28). Mpa, however, cannot facilitate robust proteasomal degradation *in vitro*. The GQYL motif is preceded by a β-grasp fold domain that tucks GQYL residues under and into the Mpa ring (34). Removal of seven N-terminal amino acids from PrcA that gate entry into 20 CPs results in “open gate” proteasomes, stabilizing the Mpa-20S CP interaction and enabling some degradation *in vitro* (21, 25, 35, 36). This activity can be further enhanced if five amino acids are added after Mpa’s β-grasp domain to help expose the GQYL motif (34). These observations strongly suggest that Mpa requires additional factors to help expose its GQYL motifs to facilitate proteolysis. Regulating Mpa engagement with 20S CPs would be a compelling hub of degradation control.

## TAG, YOU’RE IT

Ubiquitylation involves a series of enzymatic steps that can be carried out by numerous enzymes in eukaryotes (reviewed in reference 37). Ubiquitin is translated as a longer protein that must be processed to expose a C-terminal di-glycine sequence that is adenylated by “ubiquitin-activating” or “E1” enzymes and ATP. From here, numerous E2 and E3 enzymes coordinate the conjugation of ubiquitin to specific substrates or onto ubiquitin itself to form chains. Although bacteria encode ubiquitin homologs, none is known to promote proteasomal degradation. Instead, Pup targets proteins for destruction by bacterial proteasomes (5, 6). Unlike highly structured ubiquitin, Pup is largely disordered (38–40). However, when Pup interacts with flexible coiled-coil domains in Mpa, it adopts a helical structure, orienting its N-terminus into the center of an Mpa hexamer and promoting substrate delivery into the proteasome core (22). Mpa hexamers form three domains including the coiled-coil region that first engages Pup, the interdomain, and the ATPase with various associated activities (AAA) domain. The interdomain was recently shown to be essential for Pup engagement and substrate degradation (41).

**Fig 1:**
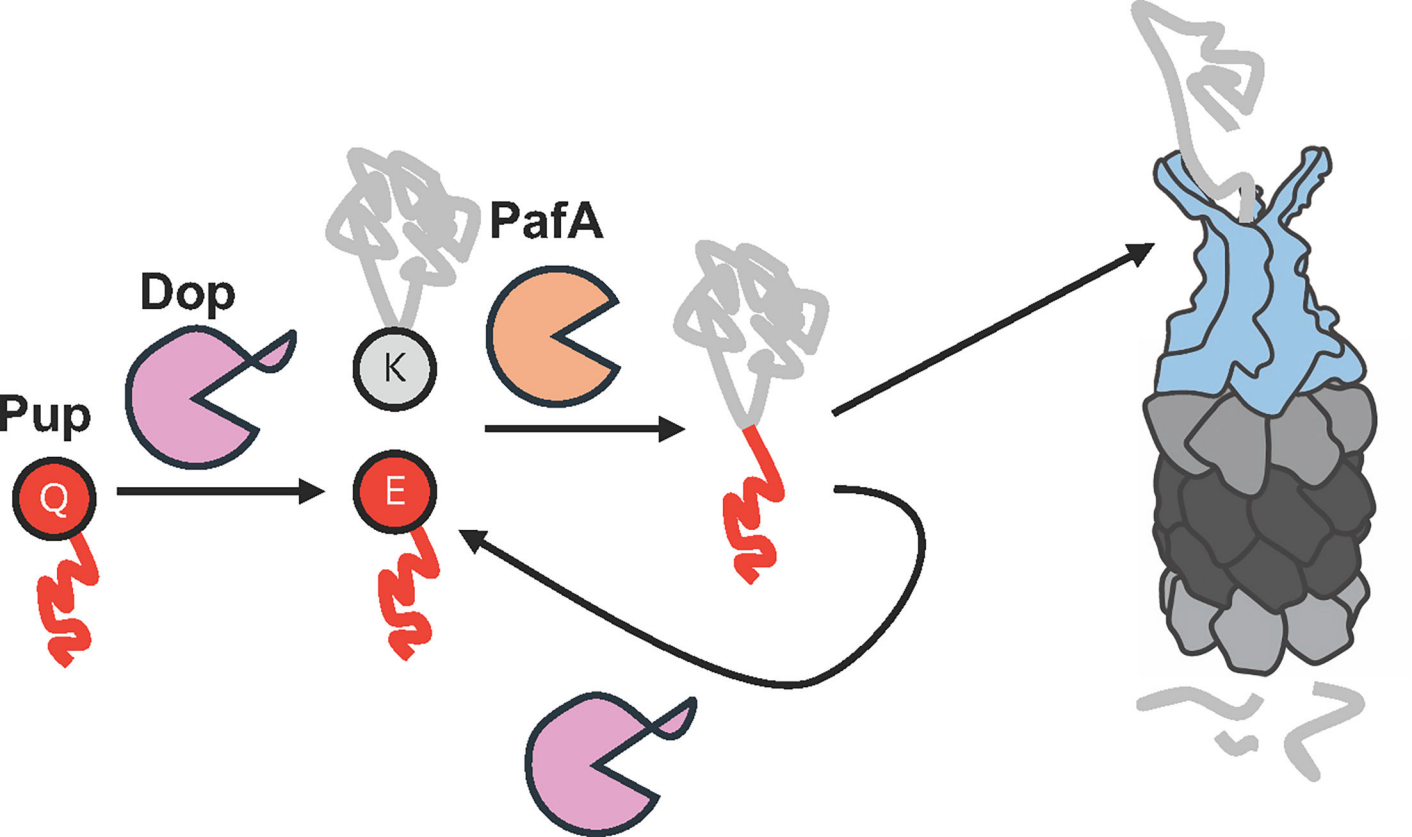
The Pup-proteasome system. In mycobacteria and other species, Pup (red) is translated with a C-terminal glutamine (Q) that must have its side chain deamidated by Dop (pink), converting this residue into a glutamate (E). PafA (orange) uses ATP to phosphorylate the side chain carboxylate of this glutamate that can be attacked by the side chain of a lysine on a targeted protein. Pup interacts with Mpa (blue), which uses ATP to unfold the pupylated protein for delivery into the proteasome core protease (dark grey). Dop also depupylates substrates, rescuing them from degradation.

In *M. tuberculosis*, Pup is translated as a 64 amino acid protein with a C-terminal glutamine that must be deamidated by deamidase of Pup (Dop) to convert it to a glutamate (42, 43) (Fig. 1). Unlike ubiquitylation, pupylation does not involve adenylation. Instead, an enzyme with structural similarity to Dop, proteasome accessory factor A (PafA), uses ATP to phosphorylate Pup’s C-terminal glutamate. This phospho-intermediate is vulnerable to nucleophilic attack by the sidechain amino group of a substrate lysine, forming an isopeptide bond (“Pup~substrate”) (44). There is no evidence of polypupylation in bacteria, although this product can be observed *in vitro* (35, 45). Overproduction of hexahistidine-tagged Pup in *M. smegmatis* results in detectable Pup~Pup linkages; however, these linkages are not attributable to specific substrates and may be a byproduct of Pup overproduction (46). Translational fusion of Pup to other proteins, including non-native proteins like GFP or endogenous proteins that are not pupylation targets, results in robust proteasome-dependent degradation (21, 45, 47), suggesting only one Pup is needed.

Pupylation is also reversible by Dop (48, 49). Deamidation and depupylation both require the breaking of an amide bond; therefore, it is presumed that the same catalytic activity of Dop is used. While the precise enzymology of this reaction is debated, Dop requires ATP, or ADP and phosphate, for catalysis (42, 48–50). One model proposes that a conserved active site aspartate directly attacks the isopeptide bond between Pup and a substrate lysine, forming a mixed anhydride bond that is hydrolyzed to release Pup from its substrate (51). An alternative hypothesis is that this aspartate and other residues coordinate phosphate and water to hydrolyze amide bonds (52, 53).

Many species encode Pup with a C-terminal glutamate (Pup_Glu_), obviating the need for deamidation and suggesting that depupylation is the primary function of Dop in these species. Moreover, Dop promotes Pup recycling. Production of Pup_Glu_ in an *M. tuberculosis dop* mutant overrides the need for Pup_Gln_ deamidation and restores pupylation; however, the pupylome can only be detected if proteasome activity is also inhibited (11). This observation suggests that in the absence of depupylation, pupylated proteins have “one-way” trips to the proteasome. In contrast, the same study showed that the same was not true for an *M. smegmatis dop* mutant expressing Pup_Glu_. However, this experiment was done under routine culture conditions, i.e., rich broth. More recently, the Gur lab showed that under starvation conditions, depupylation is crucial to maintain Pup levels (54). In another study, Zerbib et al. reported that Pup lacks favorable proteasome cleavage sites, allowing pupylated fragments to be released from proteasome core proteases *in vitro*. This report additionally showed that Dop can remove Pup from protein fragments, which can be used for new pupylation reactions, further supporting a role for Dop in Pup recycling (55). These *in vitro* studies reinforce the possibility that recycling of Pup from partially degraded proteins might occur in bacteria.

While Dop activity is essential to maintain a robust pupylome, it is not the only protein with depupylase activity: PafA can depupylate one substrate to pupylate another (50, 56). PafA has the same conserved active site aspartate found in Dop, but unlike Dop, PafA cannot deamidate Pup_Gln_. While it is unknown if the “transpupylation” activity of PafA occurs in bacteria, it represents an attractive possibility for regulating the pupylated state of a protein.

It is notable that not all pupylated proteins are always actively degraded in *M. tuberculosis* (30). This observation might be due to features such as sequence, structure, localization, or binding partners that might prevent substrate access to Mpa-capped proteasomes. Pupylation is also known to have a non-degradative role in bacterial species like *Corynebacterium glutamicum*, which encodes ARC/Mpa, Pup, PafA, and Dop, but not proteasome core subunits. Like many other bacterial species, *C. glutamicum* makes bacterioferritin (BfrB), a protein that forms multimeric, iron-storage complexes, and is a target of pupylation. Under low iron conditions, ARC disassembles pupylated BfrB complexes, releasing iron stores to promote bacterial growth (57). It is possible, if not likely, that Pup can function similarly in proteasome-bearing bacteria, but this idea remains to be determined.

## SO MANY SUBSTRATES, SO LITTLE TIME

Unlike the ubiquitin-proteasome system that includes hundreds of different enzymes, only two enzymes are responsible for all pupylation and depupylation in bacteria. Thus, understanding how numerous different proteins are selected for modification and degradation is a mystery. Some substrates are pupylated with variable efficiency *in vitro*, at least partly due to the charges of amino acids near targeted lysines (58). However, beyond the absolute requirement of a surface-exposed lysine, motifs defining which are pupylated have not been identified. Moreover, given that the expression of *M. tuberculosis pup_Glu_* and *pafA* in *Escherichia coli* is sufficient to pupylate dozens of endogenous proteins, there does not appear to be essential mycobacterial-specific factors beyond PafA required for pupylation (59).

Depupylation efficiency of specific substrates also varies *in vitro,* and steric hindrance associated with positioning pupylated substrates in the active site is hypothesized to be a contributing factor (60, 61). This steric hindrance may be affected by an approximately 40 amino acid sequence near the Dop active site dubbed the “Dop-loop” that is absent from PafA (53, 62, 63). Despite the lack of obvious structure, the loop sequence is highly conserved. Deletion of most of the loop sequence results in faster depupylation *in vitro* and yields diminished steady-state pupylome levels in *M. smegmatis* and *M. tuberculosis* (61, 64).

Small deletions of amino acids from the Dop-loop lead to an accumulation of Pup~NuoG, a component of a type I NADH dehydrogenase complex (64). Based on this observation, it was hypothesized that the Dop loop facilitates access to the NuoG pupylation site if, or when, it is occluded by other proteins in the NADH dehydrogenase complex. NuoG is not the only pupylated protein that accumulates in strains over-producing specific Dop-loop mutants, suggesting different parts of the loop are needed for access to pupylated lysines in other substrates.

**Fig 2:**
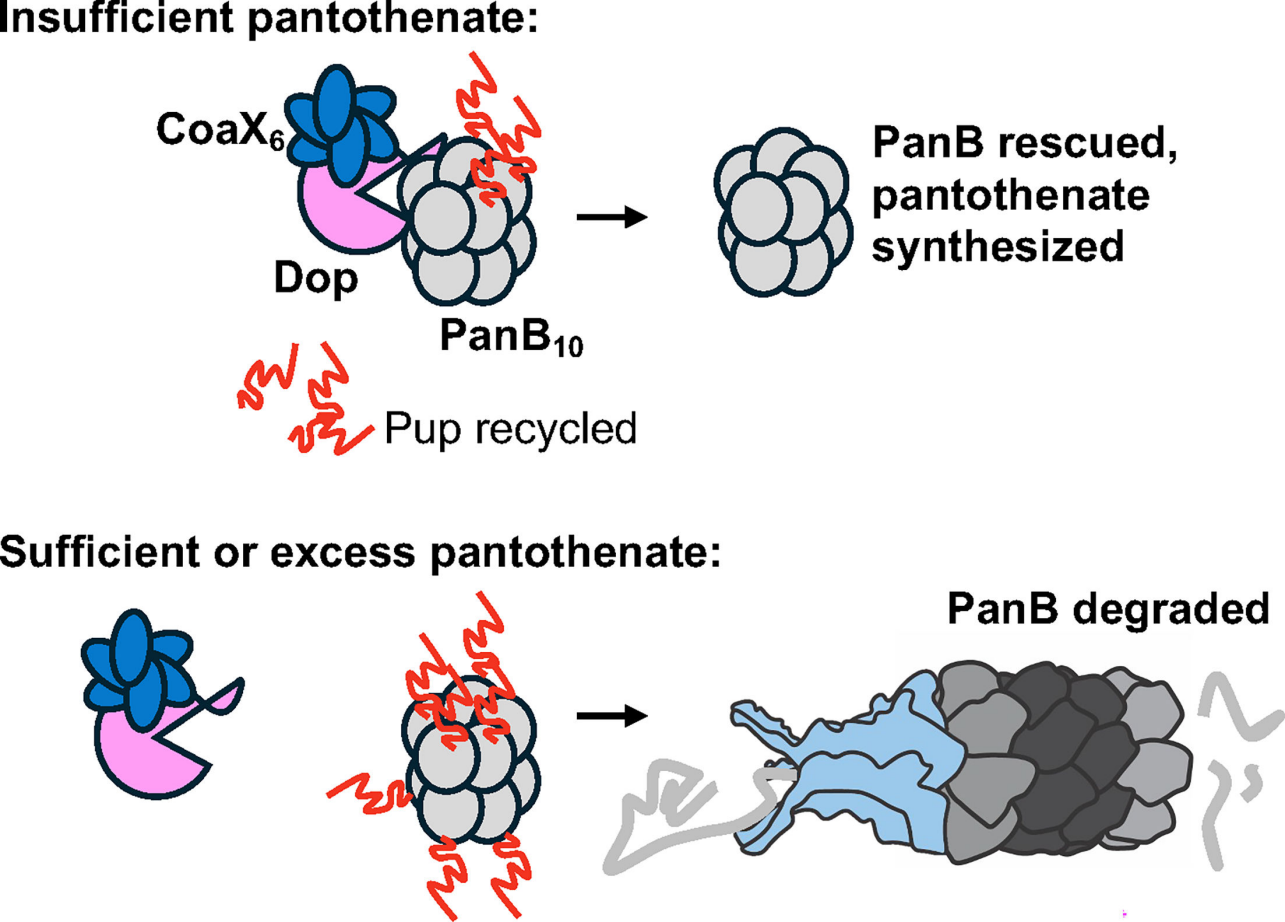
Illustration of PanB regulation. When pantothenate is insufficient, hexameric CoaX and Dop increase removal of Pup from PanB decamers, rescuing PanB and enabling pantothenate synthesis. When pantothenate is sufficient, PanB remains pupylated and is degraded by the proteasome.

A breakthrough in understanding how the stability of pupylated proteins can be dynamically regulated came through the identification of a Dop-binding protein called CoaX. A homologue of dimeric pantothenate kinases (“PanKs” encoded by *coaA* genes), *M. tuberculosis* CoaX specifically regulates depupylation of PanB, an enzyme that catalyzes an early step in pantothenate (vitamin B5) synthesis. Pantothenate is an essential precursor of coenzyme A (CoA) synthesis. For CoA synthesis, pantothenate must be phosphorylated by a PanK; in some bacterial species like *E. coli*, this process is inhibited by excess CoA, representing a classic feedback regulatory process (65). Unlike PanKs, CoaX does not function as a dimeric kinase. Instead, CoaX forms a trimer-of-dimers that interacts with Dop (63). In the absence of pantothenate, CoaX enhances Pup~PanB depupylation, rescuing PanB from proteolysis, presumably increasing pantothenate synthesis (Fig. 2, top). In the presence of pantothenate, however, CoaX-stimulated depupylation of Pup~PanB is inhibited, and PanB is degraded (63) (Fig. 2, bottom). Importantly, CoaX-Dop activity represents one of the few known regulatory mechanisms for pantothenate synthesis in bacteria, solidifying a role for proteolysis in negative feedback regulation of central metabolism.

Identification of the depupylation regulatory function of CoaX raises the possibility that the stability of other substrates might be modulated by their own depupylation adaptors in response to environmental cues. Transcription of the core Pup-proteasome system genes is not induced or repressed under any known conditions in mycobacteria, suggesting changes in activity are not due to changes in gene expression. Nonetheless, variable culture conditions can alter the steady-state pupylome in mycobacteria; for example, incubation of *M. smegmatis* in media lacking a nitrogen source results in a dramatic loss of pupylated proteins, perhaps due to increased protein turnover by proteasomes to provide nitrogen to starved bacteria (45). Similarly, in *M. tuberculosis*, Mpa-dependent proteasomal degradation is globally enhanced when forced to use nitrate as a sole nitrogen source (47). This result may indicate the existence of regulators that either increase the association of Mpa with proteasomes or enhance interactions between Mpa and pupylated proteins or increase the rate of Mpa-proteasome activity in response to environmental cues.

## OUTSTANDING QUESTIONS

**Fig 3:**
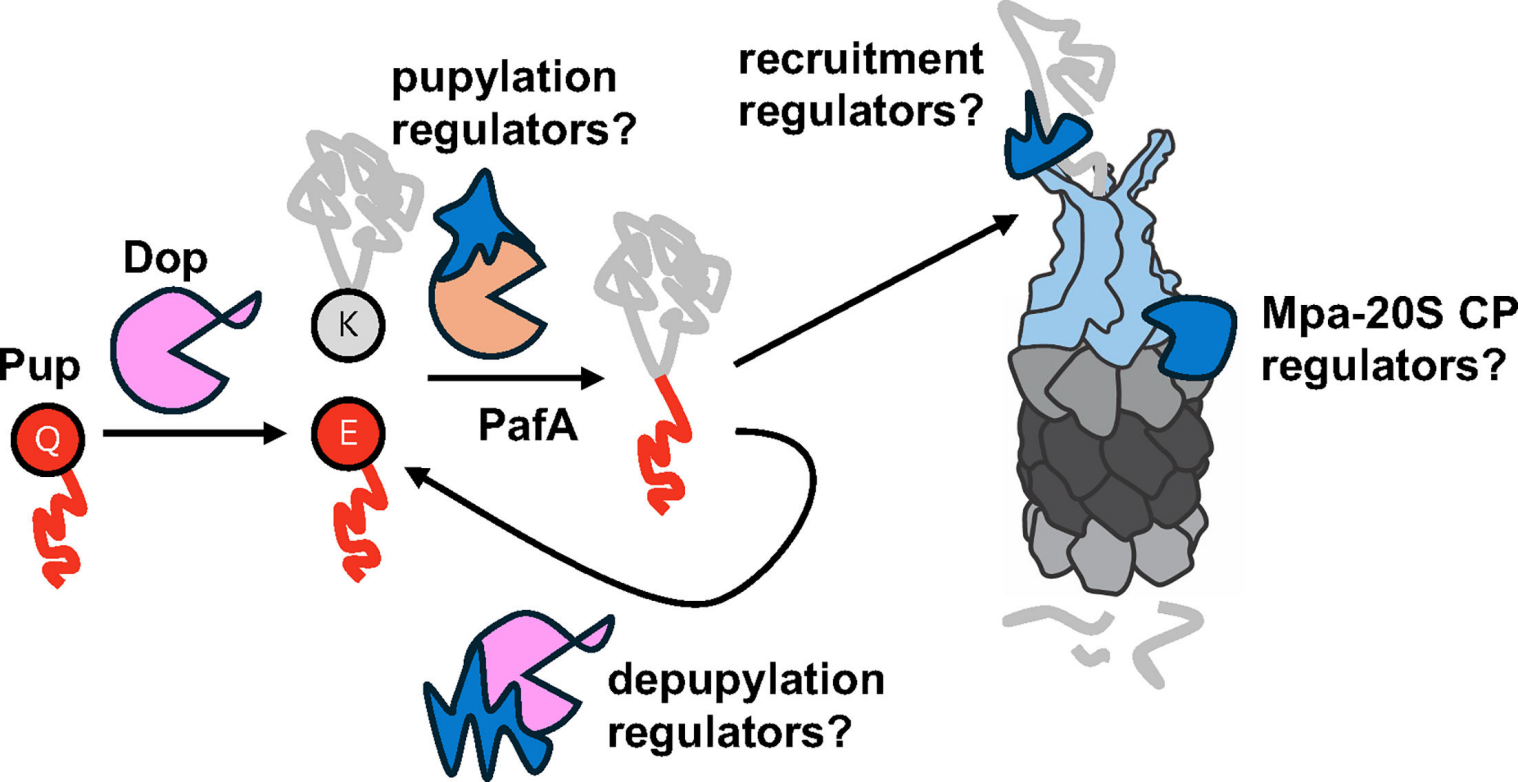
Potential regulatory mechanisms for the Pup-proteasome pathway. Proteins could interact with PafA or Dop to regulate pupylation of substrates, Mpa to regulate recruitment of proteasome substrates, and/or Mpa and the proteasome to regulate degradation directly.

We have only just begun to understand how bacteria regulate proteasomal degradation, and many exciting questions remain (Fig. 3). Exactly how the interaction between CoaX and pantothenate modulates Dop activity remains to be determined. Because CoaX specifically regulates depupylation of Pup~PanB, it is likely that CoaX affects Dop and Pup~PanB interactions (63). Pantothenate might modulate CoaX-mediated regulation by inducing conformational changes in CoaX that occlude the Dop active site or reduce interaction with Pup~PanB. The existence of CoaX has also opened the possibility that additional regulators of Dop control the stability of individual pupylation substrates, of which over 60 have been identified (30). Another possibility is that substrates themselves could affect their pupylation status. Like feedback inhibition, in which the presence of excess end product can inhibit enzyme activity, an excess of an intermediate or final product could inhibit the depupylation of the enzyme involved in its synthesis. Yet another possibility is that pupylation is an irreversible, one-way ticket to the proteasome for some proteins.

There are even more questions about PafA, a protein for which no direct regulators have been found. In addition to looking for Dop interaction partners, Kahne et al. looked for PafA-binding proteins. However, they did not find robust interactors that were not known substrates (63). This result may indicate that PafA-substrate interactions are transient, “tag-and-run” reactions. Another possibility is that PafA is localized or anchored near complexes that require stringent regulation but does not itself need tight association with targeted proteins.

Finally, nothing is known about how proteasome activator association with 20S CPs is regulated. Inhibition of proteasome activity allows both Mpa and PafE to associate tightly with 20S CPs in *M. tuberculosis*, suggesting activator association is dynamic (26). Other studies show that 20S CPs can be phosphorylated, the consequences of which are unknown (66, 67). In addition to phosphorylation, other PTMs might regulate interactions between enzymes in the proteasome system. With increased use of high-resolution mass spectrometry, future studies may find new PTMs on proteasome components and accessory factors. As in the ubiquitin field before it, we anticipate that advances in PTMs and proteasome activity will yield exciting new insights in biology.
